# Growth Trade-Offs Accompany the Emergence of Glycolytic Metabolism in Shewanella oneidensis MR-1

**DOI:** 10.1128/JB.00827-16

**Published:** 2017-05-09

**Authors:** Lon M. Chubiz, Christopher J. Marx

**Affiliations:** a Department of Biology, University of Missouri—St. Louis, St. Louis, Missouri, USA; b Department of Organismic and Evolutionary Biology, Harvard University, Cambridge, Massachusetts, USA; c Department of Biological Sciences, University of Idaho, Moscow, Idaho, USA; d Center for Modeling Complex Interactions, University of Idaho, Moscow, Idaho, USA; e Institute for Bioinformatics and Evolutionary Studies, University of Idaho, Moscow, Idaho, USA; Philipps-Universität Marburg

**Keywords:** adaptive mutations, glycolysis, metabolic capacity, substrate promiscuity, trade-offs

## Abstract

Bacteria increase their metabolic capacity via the acquisition of genetic material or by the mutation of genes already present in the genome. Here, we explore the mechanisms and trade-offs involved when Shewanella oneidensis, a bacterium that typically consumes small organic and amino acids, rapidly evolves to expand its metabolic capacity to catabolize glucose after a short period of adaptation to a glucose-rich environment. Using whole-genome sequencing and genetic approaches, we discovered that deletions in a region including the transcriptional repressor (*nagR*) that regulates the expression of genes associated with catabolism of *N*-acetylglucosamine are the common basis for evolved glucose metabolism across populations. The loss of *nagR* results in the constitutive expression of genes for an *N*-acetylglucosamine permease (*nagP*) and kinase (*nagK*). We demonstrate that promiscuous activities of both NagP and NagK toward glucose allow for the transport and phosphorylation of glucose to glucose-6-phosphate, the initial events of glycolysis otherwise thought to be absent in S. oneidensis. ^13^C-based metabolic flux analysis uncovered that subsequent utilization was mediated by the Entner-Doudoroff pathway. This is an example whereby gene loss and preexisting enzymatic promiscuity, and not gain-of-function mutations, were the drivers of increased metabolic capacity. However, we observed a significant decrease in the growth rate on lactate after adaptation to glucose catabolism, suggesting that trade-offs may explain why glycolytic function may not be readily observed in S. oneidensis in natural environments despite it being readily accessible through just a single mutational event.

**IMPORTANCE** Gains in metabolic capacity are frequently associated with the acquisition of novel genetic material via natural or engineered horizontal gene transfer events. Here, we explored how a bacterium that typically consumes small organic acids and amino acids expands its metabolic capacity to include glucose via a loss of genetic material, a process frequently associated with a deterioration of metabolic function. Our findings highlight how the natural promiscuity of transporters and enzymes can be a key driver in expanding metabolic diversity and that many bacteria may possess a latent metabolic capacity accessible through one or a few mutations that remove regulatory functions. Our discovery of trade-offs between growth on lactate and on glucose suggests why this easily gained trait is not observed in nature.

## INTRODUCTION

The ability to utilize specific carbon sources is a defining feature of many bacterial species, yet it is often unclear why a given organism cannot use more substrates than it does. In general, the catabolic potential of an organism has become increasingly predictable from annotated genome sequences (1, 2). The reconstruction of genome-scale metabolic pathways and computational frameworks, such as flux balance analysis, that seek to predict how a cell would grow optimally given its assumed metabolic network has been instrumental in making such predictions (3, 4). Many of the failed predictions for substrate use emerge from the inclusion or exclusion of reactions that are actually present (2). Another notable challenge for prediction has been substrate transport. Broad families of transporters are easily recognizable in terms of their mechanisms (ATP-driven versus proton motive force), but substrate specificity is often either hard to determine or exhibits a fair degree of promiscuity for alternative substrates (5). For example, essentially all free-living bacteria have enzymatic pathways that connect glucose to the tricarboxylic acid cycle, yet many organisms cannot grow on glucose (6). This discrepancy between metabolic predictions and experimental observations remains one of the chief limitations of genome-scale metabolic models and, by extension, is one of the primary challenges in predicting metabolic interactions in microbial communities, even for well-resolved metabolic interactions (2, 7–9).

Here, we consider the physiology and evolution of metabolic capacities that are immediately proximal to what an organism can accomplish from two primary perspectives. First, what metabolic traits must evolve to allow for the utilization of a novel compound for which only one or two enzymatic reactions are missing? Second, does the evolution of novel compound utilization engender growth trade-offs upon other substrates, such that this may prevent such adaptation from occurring in nature?

One critical driver in the evolution of new metabolic traits is the introduction of novel enzymes or transporters via horizontal gene transfer (HGT). While HGT tends to occur at the highest rates between closely related bacterial species, horizontally transferred metabolic pathways have even been observed between the domains Archaea and Bacteria (10). These events can range from involving single genes, akin to the acquisition of antibiotic resistance, to involving whole suites of metabolic pathways associated with a new lifestyle, such as pathways required for single-carbon metabolism or the degradation of xenobiotic chemicals (10–13).

A second and sometimes underappreciated process for the evolution of novel metabolism is via mutations to genes already present in an organism (14). One hurdle can be inappropriate gene regulation, whereby the bona fide enzymatic activity needed to use a new substrate already exists but is simply not expressed sufficiently. A prominent example of this type of adaptation is the emergence of citrate utilization during the course of long-term serial propagation of Escherichia coli in a minimal medium containing glucose and citric acid (15, 16). After nearly 30,000 generations, that E. coli acquired several mutations facilitating the constitutive expression of a citrate transporter gene (*citT*) (16). This mutation allows for the uptake and catabolism of citric acid under aerobic conditions—normally a metabolism used by E. coli under anaerobic conditions (16–18). An even more challenging scenario exists when both the regulation and the enzymatic specificity are inappropriate. There have been multiple examples from primary catabolism and biosynthetic reactions whereby the promiscuous activity of an enzyme—often which needed to be overexpressed in the first place—permitted the utilization of a novel substrate (19, 20). When considering these two types of adaptations to a new substrate, metabolic reconstruction will often include enzymes that turn out not to be expressed, leading to a “false positive” for substrates like citrate in E. coli, but does not include promiscuous activities, thereby generating a “false negative” for considering where changes in gene regulation can permit growth on an apparently unusable substrate. Taken together, these examples, among many others, illustrate that bacterial carbon utilization can be a very plastic phenotype, even in the absence of horizontal gene transfer.

Given the plasticity in evolving novel metabolisms that has often been observed under laboratory conditions, the question arises as to why such events have not yet occurred in nature for those systems. A primary difference may be that experimental evolution tends to be implemented in environments for which there is no change in substrates over time, such that there is no selective pressure to remain fit on alternative resources (21). Because of this, mutations that increase fitness on the novel substrate but simultaneously decrease fitness on one or more previously utilized substrates (antagonistic pleiotropy) could prevent expansion of the organism's niche to include the novel substrate in nature. Although there are examples where pleiotropy during adaptation to already utilizable resources has been synergistic rather than antagonistic (22), there are numerous other cases of antagonistic pleiotropy (23–26). What is less clear is how often antagonistic pleiotropy is involved during the adaptation to a previously unused substrate, such that it may present a reason why such niche expansion had not occurred in nature. To address these questions, we examined the genetic and biochemical bases for glucose metabolism in Shewanella oneidensis, a metabolic specialist normally unable to consume glucose.

Shewanellaceae are widely regarded as a metabolically diverse group of bacteria and are well known for their prolific use of transition metal and organic terminal electron acceptors (27, 28). Among Shewanellaceae, S. oneidensis possesses a narrow catabolic capacity limited to fermentation products, such as lactate and succinate, and not carbohydrates, such as glucose, galactose, or fructose (29, 30). However, like many other aquatic bacteria, S. oneidensis is capable of catabolizing the glucose analog *N*-acetylglucosamine (GlcNAc) (31). Based on a genome-scale metabolic reconstruction of S. oneidensis, part of this limited catabolic capacity is the result of an incomplete Embden-Meyerhof-Parnas pathway (29, 30, 32–34). S. oneidensis lacks genes encoding phosphofructokinase; however, it does have complete Entner-Doudoroff and pentose phosphate glycolytic pathways, suggesting that hexose metabolism should be possible (30, 32, 33). Additionally, a recent survey of known transport mechanisms in S. oneidensis has shown that there are only a few genes encoding a phosphotransferase system with an unknown cognate substrate(s) and a sparse array of ATP-binding cassette carbon-uptake transporters. Most carbon transport in S. oneidensis appears to be mediated through major facilitator superfamily (MFS) transporters, similar to that in many pseudomonads (29, 30, 32). In all, S. oneidensis appears to have many of the core requirements for the metabolism of complex carbon sources such as glucose, but appears to lack the early necessary steps of transport and phosphorylation.

Recently, two distinct approaches have shown that glucose metabolism is possible in S. oneidensis. First, Choi and coworkers demonstrated that S. oneidensis can be engineered to consume glucose under a wide variety of growth conditions by the introduction of a glucose transporter gene (*gluP*) and a hexokinase gene (*glk*) from Zymomonas mirabilis (35). Thus, the apparent limitations to growth on glucose in S. oneidensis are transport and phosphorylation, consistent with metabolic modeling predictions. Second, under aerobic laboratory conditions, S. oneidensis MR-1 has been reported to acquire the ability to utilize glucose as the sole source of carbon and energy (36). In both of these cases, it remains unclear what the genetic and physiological bases for hexose metabolism are and what forces, such as trade-offs, may have prevented the wild-type strain from evolving to use glucose.

In this work, we selected for mutants of S. oneidensis capable of utilizing glucose and identified the genetic basis across multiple populations as the repeated loss of a transcriptional repressor (*nagR*) that controls the expression of enzymes involved in the metabolism of GlcNAc. We show genetically and biochemically that glucose is transported and phosphorylated by the GlcNAc transporter (NagP) and kinase (NagK), respectively, which are constitutively expressed due to the loss of *nagR*. Likewise, we found via ^13^C metabolic flux measurements that glucose is oxidized through the Entner-Doudoroff pathway. Finally, we observed significant growth and yield impairments for glucose-evolved strains when grown on lactate, which is a preferred carbon source for S. oneidensis. This suggests that trade-offs may have prevented glucose metabolism from emerging in S. oneidensis in its natural environment. Collectively, these findings demonstrate that increased catabolic capacity can emerge rapidly via gene loss, a process generally associated with the deterioration of metabolic function. Likewise, bacteria viewed as having narrow carbon substrate ranges may often have the latent ability to engage in more diverse metabolisms that are mutationally accessible through just one or a few changes.

## RESULTS

### Rapid evolution of glucose catabolism is mediated through the loss of *nagR*.

To isolate S. oneidensis mutants with evolved glucose catabolism, we serially transferred four cultures (each derived from independent colonies) in a glucose-supplemented rich medium for approximately 20 generations. These cultures were then screened for mutants capable of glucose catabolism on a solid minimal medium with glucose as the sole carbon source (herein referred to as Glu^+^). From each culture, 12 isolates were subsequently grown in glucose minimal medium and verified as S. oneidensis by PCR and plate phenotyping. These mutants were then passaged at a 1:256 dilution through lactate minimal medium for one growth cycle and subsequently returned to glucose minimal medium to confirm that glucose growth was the result of a beneficial mutation and not simply from slow phenotypic acclimation to glucose as a growth substrate. We observed that the Glu^+^ phenotype in all 12 isolates from each of the four populations appeared to be genetic rather than physiological based on this assay.

**FIG 1:**
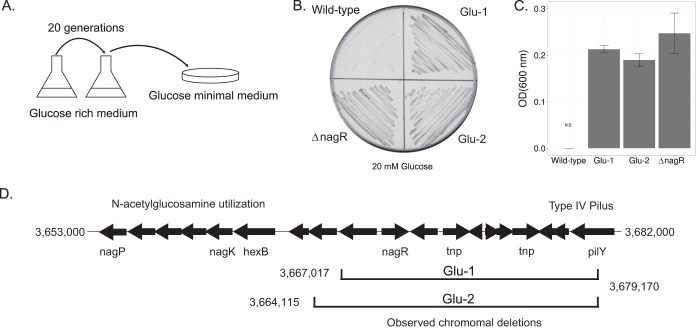
Emergence of the Glu^+^ phenotype is a result of losing *nagR* via deletion. (A) Experimental procedure for selection of Glu^+^ mutants. (B) Growth of wild-type, Glu-1, Glu-2, and Δ*nagR* isolates on minimal medium agar plates supplemented with 20 mM glucose as a carbon source at 30°C for 72 h. (C) Yields of wild-type, Glu-1, Glu-2, and Δ*nagR* isolates in liquid minimal medium supplemented with 20 mM glucose after 48 h of growth. (D) The *N*-acetylglucosamine utilization cluster and chromosomal regions lost, as identified by whole-genome sequencing and analysis with the breseq pipeline. Annotations from the left: *nagP*, GlcNAc permease; *nagK*, GlcNAc kinase; *hexB*, β-*N*-acetylhexosaminidase; *nagR*, GlcNAc operon repressor; *tnp*, ISSod1 transposase; and *pilY*, type IV pilus protein.

To find the causative beneficial mutation(s) in these mutants, we performed whole-genome sequencing of two independently derived Glu^+^ isolates. Putative mutations were identified using the breseq sequence analysis pipeline developed by Barrick and coworkers (37), yielding two candidate mutations by comparison with the wild-type S. oneidensis. The most promising was a deletion spanning a chromosomal region containing predicted open reading frames (ORFs) annotated as a type IV pilus apparatus, an NADH dehydrogenase gene, and a transcriptional repressor (*nagR*) believed to be involved in regulating *N*-acetylglucosamine (GlcNAc) utilization (Fig. 1D), as it was present in both sequenced isolates and was near known GlcNAc utilization genes. Confirming the independent provenance of these two isolates, the span of the deletion differed in the proximal end of each deletion (Fig. 1D). Examining all 48 isolates by PCR genotyping, we found that they all contained deletions spanning *nagR*. Examining the novel junctions identified by breseq does not appear to indicate any strong homology that would facilitate efficient homologous recombination. We do note that the region lost contains two copies of the ISSod1 transposon, which may have been involved in promoting the loss of this common region of DNA.

**FIG 2:**
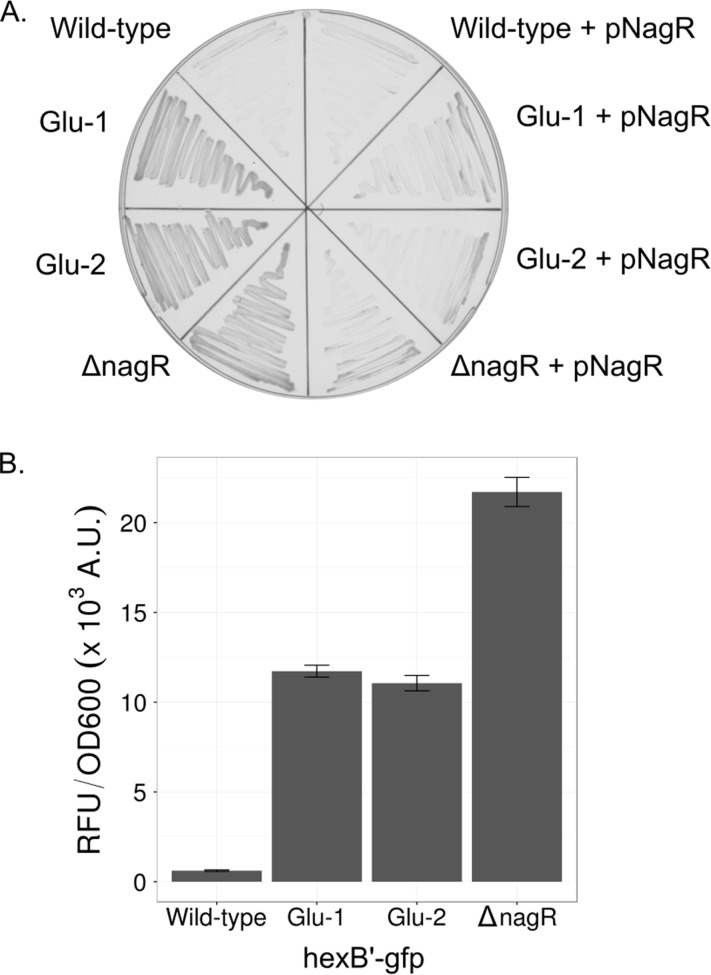
Loss of *nagR* results in constitutive expression of *N*-acetylglucosamine utilization genes. (A) Wild-type, Glu-1, Glu-2, and Δ*nagR* isolates harboring either pBRR-MCS-2 or pNagR grown on minimal medium agar plates supplemented with 20 mM glucose and 50 μg/ml kanamycin at 30°C for 72 h. (B) Green fluorescent protein expression from the NagR-repressed *hexB* promoter in the wild type and Glu-1, Glu-2, and Δ*nagR* mutants, as determined by measurement of emission at 520 nm and normalized to the OD_600_ of the culture. Cells were grown to mid-log phase in liquid minimal medium supplemented with 20 mM dl-lactate.

Based on the chemical structural similarity of glucose to GlcNAc, we suspected the loss of *nagR* and the subsequent use of constitutively expressed GlcNAc catabolic genes as a possible source of the Glu^+^ phenotype. To confirm this hypothesis, we deleted *nagR* in the wild-type background and observed the subsequent emergence of the Glu^+^ phenotype with final yields similar to those of the evolved Glu^+^ mutants (Fig. 1C). Likewise, the complementation of *nagR* in either the *nagR* deletion strain or the evolved mutants abolished the ability of S. oneidensis to grow on glucose (Fig. 2A). These results clearly point to the loss of *nagR* as being critical for the evolved glucose catabolism.

### Loss of *nagR* results in constitutive expression of the *nag* regulon.

Using a green fluorescent protein (GFP) transcriptional reporter, we found that NagR-repressed genes are constitutively expressed in Glu^+^ mutants as well as in a synthetic *nagR* deletion mutant. NagR has been characterized as a LacI family transcriptional repressor and is suspected to bind numerous DNA binding sites within the chromosomal neighborhood of the *nagR-nagP* gene cluster (31, 38). Using these bioinformatics-based predictions, we hypothesized that the loss of *nagR* was ultimately leading to unregulated constitutive expression of NagR-repressed genes. To test this, we constructed a transcriptional fusion of the promoter region of *hexB* (which encodes a β-*N*-acetylhexosaminidase) to *gfp* in the broad-host-range vector pPROBE-gfp (39). The promoter driving *hexB* has been suggested to also drive expression of the distal GlcNAc transport and catabolic genes (38). All *hexB* fusion plasmid-harboring strains were grown in lactate minimal medium to allow for the growth of the mutants and the wild type. In congruence with our suspected mechanism, we observed 6- to 12-fold higher levels of GFP expression in the *nagR* and Glu^+^ mutants than in the wild type. Interestingly, we also observed a 1.85- to 1.96-fold increase in GFP expression in the synthetic *nagR* background over that in the Glu^+^ mutants (Welch *t* test, *P* < 0.001). A possible explanation is that the impaired growth on lactate (see Fig. 6; see also Fig. S2 in the supplemental material) observed in evolved and synthetic *nagR* mutants altered the rates of transcriptional initiation or the plasmid copy number, with the synthetic *nagR* deletion displaying the greater effects. Regardless, these data indicate that constitutive activation of *nagR*-regulated genes is likely the regulatory mechanism yielding Glu^+^ mutants.

### Uptake of glucose in Glu^+^ mutants is mediated by NagP.

The fundamental limitation for glucose utilization by S. oneidensis is believed to stem from the lack of both a glucose-specific transport mechanism and a glucokinase needed to produce glucose-6-phosphate (29). Notably, the S. oneidensis MR-1 genome does encode a number of carbohydrate transport mechanisms (32). However, these genes have either become pseudogenes as a result of frameshift mutations and/or insertions or do not encode for proteins functionally capable of transporting glucose (29). Recently, it was demonstrated that these limitations can be circumvented via heterologous expression of a glucose transporter gene (*gluP*) and a glucokinase gene (*glk*) from Zymomonas mirabilis, allowing for growth on glucose (35); thus, supporting a model in which both transport and glucose phosphorylation are limiting events. To understand how S. oneidensis overcomes these limitations via the loss of *nagR*, we explored the role of the GlcNAc transporter (*nagP*) in glucose catabolism.

**FIG 3:**
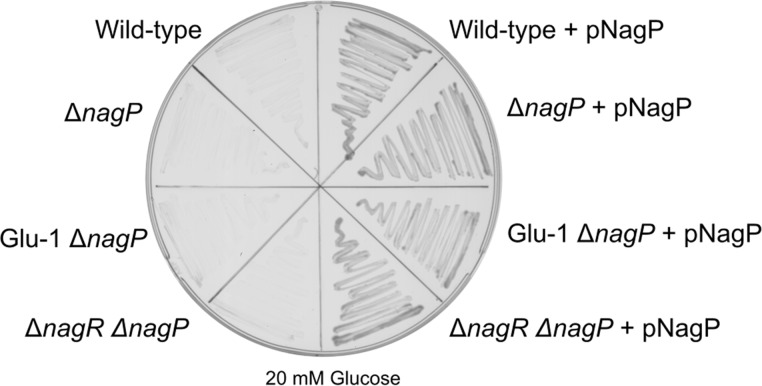
Expression of the *N*-acetylglucosamine transporter, NagP, is sufficient to allow growth on glucose. Wild-type, Δ*nagP*, Glu-1 Δ*nagP*, and Δ*nagR* Δ*nagP* isolates harboring pBRR-MCS-2 or pNagP grown on minimal medium agar plates supplemented with 20 mM glucose and 50 μg/ml kanamycin at 30°C for 72 h.

As previously demonstrated, the loss of *nagR* results in constitutive expression of genes involved in GlcNAc uptake and catabolism, including *nagP*, encoding a GlcNAc permease. Based on the structural similarity of GlcNAc and glucose, we hypothesized that NagP may allow promiscuous glucose transport and obviate the first barrier to glucose utilization. To determine the role of NagP in facilitating glucose catabolism, we created a *nagP* deletion mutant and tested its growth on glucose. We found that the loss of *nagP* results in a loss of the Glu^+^ phenotype in both the spontaneous mutants and in the *nagR* mutant (Fig. 3). Additionally, complementing *nagP* restored the growth on glucose. Interestingly, the expression of *nagP* in the wild-type background (i.e., providing only a mechanism of glucose transport) was sufficient to allow for growth on glucose. Taken together, these data show that the loss of *nagR*, and thus the overexpression of *nagP*, is sufficient to facilitate glucose catabolism in S. oneidensis. Likewise, our observations show that one or more enzymes must be displaying glucokinase activity to convert glucose to glucose-6-phosphate.

### Glucose can be phosphorylated by the GlcNAc kinase, NagK.

**FIG 4:**
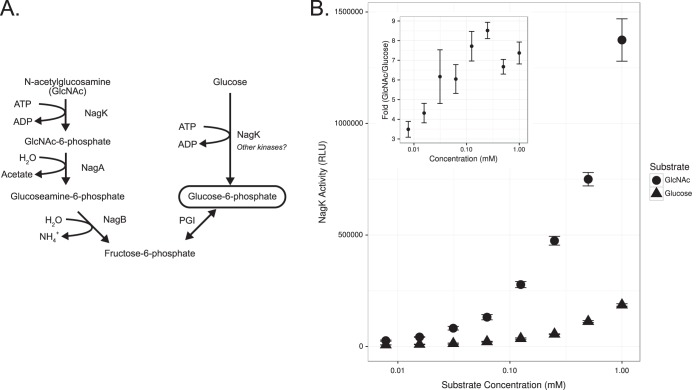
The kinase (NagK) of the *N*-acetylglucosamine pathway is capable of converting glucose to glucose-6-phosphate. (A) The *N*-acetylglucosamine pathway for conversion of GlcNAc and glucose to glucose-6-phosphate. (B) *In vitro* kinase activities of purified NagK with GlcNAc and glucose substrates and the fold differences of activities between these substrates across the same concentrations (inset).

In addition to the transport by NagP, we tested whether the GlcNAc kinase, NagK, was capable of converting glucose to glucose-6-phosphate. Despite *nagP* expression having been sufficient for the Glu^+^ phenotype, the phosphorylation of glucose to glucose-6-phosphate still presented a source of uncertainty in fully characterizing glucose catabolism in Glu^+^ mutants. Based on the genes that were found to be constitutively expressed upon the loss of *nagR* and that the complementation of *nagR* repressed glucose catabolism, we hypothesized that the source of glucokinase activity may be the overexpression of *nagK*, a GlcNAc kinase gene. To test this possibility, we performed kinase activity assays with purified NagK to determine whether NagK is able to phosphorylate glucose in addition to its known substrate, GlcNAc (Fig. 4A). We found that, across a wide range of concentrations (0.1 to 1 mM), NagK is capable of phosphorylating glucose at 3.5- to 8.4-fold lower levels of activity than for GlcNAc (Fig. 4B). While additional kinases may phosphorylate glucose, these data strongly indicate that NagK is capable of preparing glucose for glycolysis.

### Glucose catabolism is mediated through the Entner-Doudoroff pathway.

After establishing a mechanism for the transport and phosphorylation of glucose, we sought to identify what glycolytic pathways are used by S. oneidensis to catabolize glucose. In many bacteria, such as E. coli, there are three canonical pathways for glucose catabolism: the Embden-Meyerhof-Parnas (EMP) pathway, the pentose phosphate (PP) pathway, and the Entner-Doudoroff (ED) pathway. The utilization of each pathway is often variable depending on substrate availability, on growth rate, and/or on adaptation to the environment (40–42). However, many bacteria lack one or more of these pathways and can rely exclusively on the EMP, PP, or ED pathway for glycolytic activity. Despite its apparent lack of carbohydrate transport capabilities, S. oneidensis possesses nearly all of the enzymes required for the EMP, PP, and ED pathways, with the notable exception of phosphofructokinase. Based on genome-scale metabolic reconstruction, the lack of phosphofructokinase is believed to result in an incomplete and inactive EMP pathway in S. oneidensis (29, 30, 33). However, there have been no empirical results to directly support this prediction, as the possibility of a sugar kinase with relaxed specificity toward fructose-6-phosphate cannot be excluded. For instance, E. coli relies on phosphofructokinase-1 (encoded by *pfkA*) for more than 90% of the conversion of fructose-6-phosphate to fructose-1,6-bisphosphate, yet it possesses a second structurally unrelated phosphofructokinase (encoded by *pfkB*) that can allow for the use of the EMP pathway in *pfkA* mutants (43, 44).

To explore how glucose is metabolized by S. oneidensis, we performed a metabolic flux analysis based on the distribution of ^13^C incorporation into protein-bound amino acids during growth on either single-carbon labeled [1-^13^C]glucose or universally labeled [U-^13^C]glucose as determined by gas chromatography-mass spectrometry (45). Batch cultures of S. oneidensis were grown in either 100% [1-^13^C]glucose or 20% [U-^13^C]glucose (the remaining 80% of the glucose was unlabeled). Mass distributions of ^13^C incorporation were used by the biochemical analysis software FiatFlux (46) to estimate the fractional contributions to key metabolites, such as pyruvate, phosphoenolpyruvate, and serine, from the major glycolytic pathways and the tricarboxylic acid (TCA) cycle.

**FIG 5:**
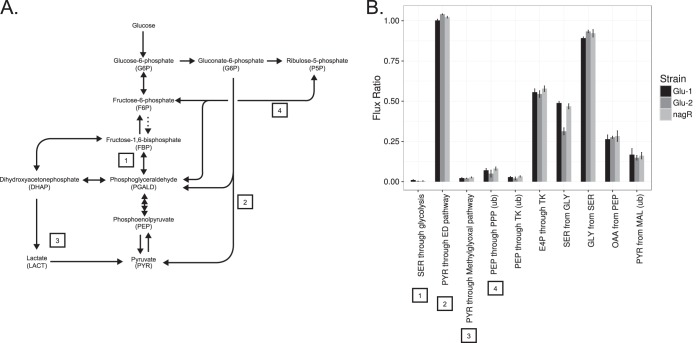
Measured flux ratios indicated that the Entner-Doudoroff pathway is the primary pathway for glucose catabolism. (A) The glycolytic pathways detectable by ^13^C-glucose labeling include the Embden-Meyerhof-Parnas pathway (1), the Entner-Doudoroff pathway (2), the methylglyoxal pathway (3), and the pentose phosphate pathway (4). (B) Measured flux ratios for major glycolytic pathways and ancillary metabolic branch points as determined by ^13^C labeling and METFoR analysis. Data and associated standard errors are from three biological replicates.

An analysis of ^13^C incorporation during growth on [1-^13^C]glucose indicated carbon flux was channeled predominantly through the ED pathway (100% ± 3%, 104% ± 3%, and 102% ± 2% for Glu-1, Glu-2, and Δ*nagR* mutants, respectively) (Fig. 5B), as estimated from the fractions of pyruvate derived from the ED pathway versus the PP or EMP pathways. In accordance with the predictions, S. oneidensis did not have any appreciable flux through the EMP pathway (1% ± 0.57%, 0.3% ± 0.1%, and 0.4% ± 0.43% for Glu-1, Glu-2, and Δ*nagR* mutants, respectively) based on the fractions of pyruvate derived from the EMP pathway. While we cannot statistically reject trace EMP pathway usage, we suspect that these values, in conjunction with the 5% to 8% of phosphoenolpyruvate derived from the PP pathway (based on [U-^13^C]glucose labeling), could be artifacts of gluconeogenic anabolic reactions. Thus, the primary mode of glycolytic activity in S. oneidensis is mediated through the ED pathway, consistent with observations made in other bacteria lacking phosphofructokinase activity (47).

### Emergence of glucose catabolism reduces the growth rate on lactate.

To gain insight into why such rapid evolution of glucose utilization had not occurred in nature, we tested whether there were any substantial decreases of fitness that may have occurred as a result of Glu^+^ mutations. Adaptation to a novel environment frequently comes after the loss of fitness or deteriorated function in other environments (25). Commonly, the timeline for observing metabolic deterioration is hundreds or thousands of generations (22, 48). Here, we desired to understand if any substantial loss of fitness or deterioration of function may have occurred as a result of Glu^+^ mutations. We examined the growth on lactate and on GlcNAc as two representative carbon sources that could be consumed by S. oneidensis prior to the selection for Glu^+^ mutants.

**FIG 6:**
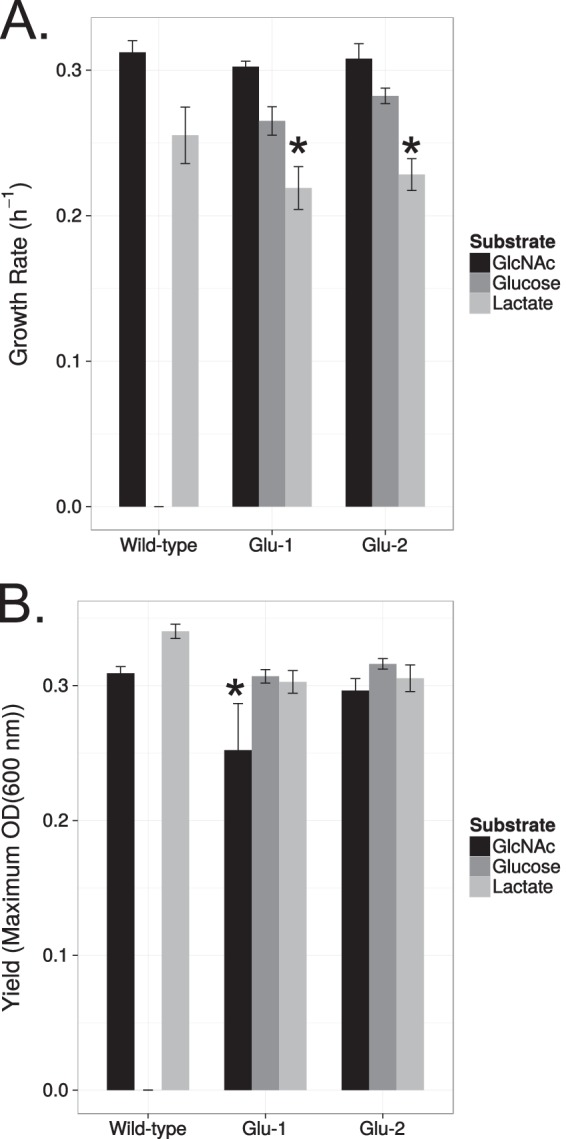
Growth and yield trade-offs exist for glucose utilization. Growth rates (A) and yields (B) for the wild type and the spontaneous Glu^+^ mutants Glu-1 and Glu-2 grown on GlcNAc (10 mM), glucose (10 mM), and dl-lactate (20 mM). Data and associated 95% confidence intervals are from 8 biological replicates. *, *P* < 0.01 versus the wild type by Wilcoxon rank sum test.

We cultured wild-type and two Glu^+^ (Glu-1 and Glu-2) isolates in minimal medium containing lactate, GlcNAc, or glucose and measured the growth rates and yields. For all cultures, no change in doubling time was observed with growth on GlcNAc due to an adaptation to growth on glucose, and both Glu-1 and Glu-2 mutants had similar growth rates on glucose. By contrast, both Glu-1 and Glu-2 mutants had significant decreases in the growth rates on lactate. Specifically, Glu-1 and Glu-2 mutants had 14.2% and 10.6% decreases, respectively, in the growth rates on lactate compared with that of the wild type (Wilcoxon rank sum test, *P* = 0.003 and *P* = 0.007, respectively) (Fig. 6A). Correspondingly, we also observed 10.8% and 10.3% average decreases in yields for Glu-1 and Glu-2 mutants, respectively, compared with that of the wild type (Wilcoxon rank sum test, *P* = 0.00015 for both cases) (Fig. 6B). A similar trend was observed for the *nagR* mutant, further implicating the loss of *nagR* as the genetic basis for this observed trade-off for growth on lactate (Fig. S2). Interestingly, we did observe that the Glu-1 mutant also had an 18.4% decrease in yield on GlcNAc compared with that of the wild type (Wilcoxon rank sum test, *P* = 0.00015) (Fig. 6B), whereas no significant decrease in yield on GlcNAc was observed for the Glu-2 mutant (Wilcoxon rank sum test, *P* > 0.5) (Fig. 6B). These data demonstrate that both growth rate and yield trade-offs exist due to mutations that allow for growth on glucose.

## DISCUSSION

In this work, we looked to explore two questions that revolve around the capacity of S. oneidensis to evolve the ability to utilize glucose. First, what metabolic traits must evolve to facilitate this novel metabolism? Second, are there growth trade-offs associated with the evolution of these metabolic traits that may explain the lack of this metabolism in the organism's natural environments? Addressing the first of these questions, we found that a single mutational step was used repeatedly by S. oneidensis to overcome the two fundamental limitations to hexose metabolism that are predicted by genome-scale metabolic models, namely, transport and phosphorylation. This single mutation is the deletion of a transcriptional regulator of GlcNAc metabolism (*nagR*) that resulted in constitutive expressions of *nagP* and *nagK*, encoding a transporter and kinase, respectively. Using genetic and biochemical approaches, we found that the expression of *nagP* is sufficient to facilitate glucose metabolism and that the GlcNAc transporter and kinase use glucose as a substrate. Additionally, we dissected how glucose is metabolized by S. oneidensis after evolutionary adaptation and observed that glucose is oxidized via the ED pathway to near exclusion, despite an intact PP pathway and near-complete EMP pathway. Thus, it appears that altering the regulation of GlcNAc metabolic genes and the reliance on their intrinsic enzymatic promiscuity are both required for engendering the wild-type S. oneidensis with the ability to consume glucose. Considering the second question, we discovered that the adaptation to metabolize glucose imposes a significant fitness trade-off in the form of decreased growth rate and yield on lactate, and for one case, a decreased yield on GlcNAc, both of which are ancestral substrates for S. oneidensis. Taken together, these findings illustrate that changes in gene regulation and underlying enzymatic promiscuity may be important mechanisms for increasing metabolic capacity, but that the associated fitness trade-offs of a new metabolism may be what prevents it from emerging in natural environments.

A more broadly framed question from these findings is why does glucose metabolism not rapidly emerge in other metabolically restricted bacteria? Most free-living bacteria have intact anabolic pathways for gluconeogenesis and, by extension, possess most, if not all, of the enzymes required for glucose catabolism via either the EMP, PP, or ED pathways (6, 49). For instance, Methylobacterium extorquens AM1 (a methylotrophic bacterium specializing in the consumption of single-carbon compounds) possesses complete PP and ED pathways, yet is notably unable to consume glucose or other carbohydrates (50, 51). One reason for this discrepancy may be the lack of a suitable transport mechanism. While S. oneidensis possesses a sufficiently promiscuous transporter in the form of NagP to allow glucose uptake, M. extorquens may not have a carbohydrate transporter with sufficiently broad specificity to facilitate the same process. Accordingly, multiple attempts to evolve glucose utilization in wild-type M. extorquens have not been successful (C. J. Marx and S. Stolyar, unpublished data).

Another hindrance to the rapid adaptation of bacteria to novel carbon sources is that enzymes capable of performing the necessary promiscuous metabolic reactions may not be adequately expressed. The work by Hall and coworkers exploring evolved β-galactosidase activity in *lacZ* mutants of E. coli illustrates this point (19). In a number of lactose-utilizing isolates, it was found that, while underlying mutations in the evolved β-galactosidase (EbgA_4_C_4_) were sufficient for enzymatic activity toward lactose, growth on lactose was only observed when a corresponding loss of function in the repressor of *ebgAC* expression (EbgR) was also present, in so-called class I mutants (19). In these class I mutants, the loss of EbgR function resulted in the constitutive expression of *ebgAC*, allowing nearly 5% of the soluble protein in E. coli to be comprised of evolved β-galactosidase and robust growth on lactose in the absence of LacZ (52). Similarly, we have found that the underlying promiscuity of NagP and NagK toward glucose is masked by NagR repression, and only on loss of NagR function did we observe growth on glucose. Akin to the studies performed on evolved β-galactosidase, it remains to be seen what the breadth of carbon sources are that Glu^+^ mutations will facilitate in S. oneidensis and how the activities of NagP and NagK may be shaped by subsequent selection in a glucose environment. Thus, the presence and adequate expression of promiscuous enzymes may be key limitations to rapid metabolic diversification in many bacteria.

Finally, if adaptation (or reversion) to glucose metabolism occurs so readily for S. oneidensis, why did adaption to catabolize glucose not occur readily in nature? Furthermore, what could have made the loss of glucose catabolic function beneficial in the first place (if indeed S. oneidensis was once able to consume glucose)? The answer to these questions may be that the loss of glucose uptake afforded S. oneidensis with improved growth on organic acids, such as lactate. From our findings, it is clear that the restoration of glucose uptake via the loss of *nagR* imposes substantial trade-offs in growth rate and yield while growing on lactate. Given the catabolic diversity in other Shewanella species and the large number of pseudogenes in carbohydrate transporters in S. oneidensis (29), it seems quite likely that carbon-diet restriction to organic and amino acids by S. oneidensis is a relatively new event in evolutionary terms and that the ancestor of modern S. oneidensis may have had a more diverse catabolic capacity. By this logic, the initial loss of glycolytic metabolism may have improved the growth rate on organic acids, while the adaptation (or reversion) to consume glucose may have been selected against due to the reduction in growth rate (27, 53). In this way, the current specialization of S. oneidensis may have arisen from a trade-off that produced a more optimal phenotype on more oxidized sources of carbon that are likely in greater abundance in its carbohydrate-poor, microaerobic aquatic environment.

Bacterial gains of metabolic function are frequently associated with the acquisition of new genetic material via horizontal gene transfer. In our study and many others, such as those described above, it is clear that the intrinsic, latent metabolic capacity of many bacteria is an underappreciated mechanism of expanding the range of carbon sources that can be used. Thus, furthering our understanding of how horizontally transferred and latent mechanisms are functionally used in natural environments will be critical to our ability to assess the ecological roles of various bacteria and to dissect what types of metabolic interactions may be functionally important in complex microbial communities. With this in mind, as the application of diverse bacteria becomes more prevalent in biotechnology, the exploration of latent metabolic capacities may be a key to expanding the possible carbon feedstocks available for use in a given process. For example, rather than focusing on heterologous expression of transporters and pathways—which requires significant optimization—a bacterium may already have a route for uptake and metabolism that can be readily selected for, as we observed here.

## MATERIALS AND METHODS

### Bacterial strains, media, and growth conditions.

All strains of Shewanella oneidensis MR-1 and Escherichia coli used in this work are described in Table S1 in the supplemental material. Routine culturing of S. oneidensis and E. coli was performed in Luria-Bertani broth liquid and solid media (10 g/liter tryptone, 5 g/liter yeast extract, 5 g/liter NaCl, and 16 g/liter agar for solid medium). The growth of S. oneidensis using glucose or dl-lactate as a carbon source at a specified concentration was carried out using a HEPES-buffered medium similar to that described by Deutschbauer and colleagues (30 mM HEPES-KOH, 1.5 g/liter NH_4_Cl, 1.75 g/liter NaCl, 0.1 g/liter KCl, 0.61 g/liter MgCl_2_, and 0.6 g/liter NaH_2_PO_4_, pH 7.2) supplemented with Wolfe's vitamins and minerals (54). Conjugal matings were performed on Luria broth agar plates supplemented with 300 μM 2,6-diaminopimelic acid to facilitate the growth of E. coli donor strains. For the selection of plasmids or chromosomal insertions, kanamycin was used at 50 μg/ml or chloramphenicol was used at 10 μg/ml, respectively. Sucrose counterselection was performed using tryptone-yeast extract (TY) agar plates (10 g/liter tryptone, 5 g/liter yeast extract, 16 g/liter agar) supplemented with 5% sucrose. All cultures were grown at 30°C, unless otherwise specified.

### Isolation of glucose-utilizing mutants.

Four independent colonies of strain S1419 (55) were each inoculated into 10 ml of Luria broth supplemented with 20 mM glucose in 50-ml Erlenmeyer flasks. Cultures were grown to saturation under well-aerated conditions (shaking at 220 rpm). Saturated cultures were then diluted 128-fold (allowing ∼7 cell generations to reach saturation) and grown to saturation under the same conditions. This procedure was repeated once more, yielding approximately 20 generations in total, and 10^−4^ dilutions of each culture were plated on glucose minimal medium agar plates. Plates were incubated for 4 days until glucose-utilizing colonies were identified. From each of the four populations, 12 isolates were collected (48 total) and were grown to saturation in glucose minimal medium. These 48 isolates were then subcultured (1:256) in dl-lactate minimal medium to saturation and subsequently subcultured (1:256) in glucose minimal medium. Lack of growth under this regime would indicate glucose utilization may have arisen from acclimation and not from an adaptive mutation. All 48 isolates grew under these conditions, whereas S1419 did not.

### Identification of mutations via whole-genome sequencing.

For whole-genome sequencing, isolates from two of the four populations were chosen. Genomic DNA was harvested by cetyltrimethylammonium bromide (CTAB)-phenol organic extraction (56), and sequencing libraries were prepared using either Illumina TruSeq reagents or in-house transpososome tagmentation (57). Sequencing reactions were performed on an Illumina HiSeq2000 or MiSeq using 50-bp single-end reads or 25-bp paired-end reads, respectively. Read processing, alignment, and mutation identification were performed using breseq version 0.21 (37). After comparison to mutations in our wild-type strain, putative Glu^+^ mutations were verified by PCR and/or capillary DNA sequencing. DNA sequencing reads have been deposited in the NCBI Sequence Read Archive (SRA).

### Creation of knockout plasmids and mutants.

All DNA oligonucleotides used in molecular cloning are described in Table S2. Deletions of *nagR* and *nagP* were performed using an “in-out” sucrose counterselection approach. As ColE1 and pBR322 plasmids replicate in S. oneidensis, we generated a new suicide integration vector, pLC284a, by PCR amplifying the *cat-sacB* cassette from pCM433 (58) and ligating this with the MluI fragment of pKNOCK-Km (59) containing the RP4 transfer origin and R6K replication origin. The *nagR* and *nagP* deletion plasmids, pLC315 and pLC311, respectively, were created by PCR amplifying two regions flanking *nagR* or *nagP* followed by isothermal DNA assembly (60) with a pLC284a plasmid that had been linearized with NotI and SalI. These plasmids were harbored in E. coli strain WM3064 to facilitate high-frequency conjugal transfer to S. oneidensis recipients. Primary insertion of these plasmids into *nagR* or *nagP* was identified by selection on Luria agar supplemented with chloramphenicol, and excision was selected for on TY agar supplemented with 5% sucrose. Deletions in sucrose-resistant mutants were verified by PCR, checking for both the expected deletion and the loss of the pLC284a backbone.

Complementation of *nagR* and *nagP* was accomplished by cloning *nagR* and *nagP*, with their respective ribosome binding sites and start codons, into the *lacZα* coding region of pBBR-MCS-2 (61), allowing for constitutive expression from the *lac* promoter. DNA fragments containing *nagP* and *nagR* flanked by EcoRI and XbaI restriction sites were generated by PCR amplification and were then cloned into the EcoRI and XbaI sites of pBBR-MCS-2 to produce plasmids pLC319 (pNagP) and pLC321 (pNagR), in that order.

### Measurement of NagR-dependent transcriptional regulation.

Changes in NagR-dependent transcriptional repression were determined using a green fluorescent protein gene (*gfp*) fusion to the promoter preceding the *hexB* gene (Fig. 1). The *hexB* promoter region was PCR amplified and cloned into the KpnI and EcoRI restriction sites of the promoter fusion vector pPROBE-gfp (39) to yield pLC307. To measure GFP expression, cells carrying pPROBE-gfp or pLC307 were grown in minimal medium containing 20 mM dl-lactate to an optical density at 600 nm (OD_600_) of 0.3 followed by fluorescence measurement using a Tecan Safire2 plate reader with an excitation of 488/5 nm and emission detection at 520/5 nm. Fluorescence values were blanked using pPROBE-gfp as a baseline followed by normalization to the OD_600_ of the sample to correct for differences in cell number.

### Purification of NagK and kinase activity assays.

NagK was purified using nickel(II)-nitrilotriacetic acid (Ni-NTA) affinity chromatography. The *nagK* coding region lacking a start codon was PCR amplified and cloned into the BamHI and PstI sites of plasmid pQE80L to produce pLC318, containing an in-frame fusion of 6 histidine codons to the N-terminal region of *nagK*.

For expression of 6×His-NagK, pLC318 was transformed into the protein expression E. coli strain ER2523 (New England BioLabs). The transformant was cultured overnight in Luria broth and subcultured 1:100 in 250 ml of fresh Luria broth and grown with vigorous aeration in a baffled 1-liter Erlenmeyer flask at 37°C. Once the OD_600_ of the culture reached 0.8, protein expression was induced by introducing isopropyl-β-d-1-thiogalactopyranoside to the culture at a final concentration of 1 mM. The induced culture was further incubated for 3 h and harvested by centrifugation at 6,000 × *g* at 4°C. The resulting cell pellet was washed once with tris-buffered saline (TBS; 50 mM Tris-HCl, 150 mM NaCl, pH 7.5), was pelleted at 6,000 × *g*, and then was frozen at −75°C. Pellets were then resuspended in TBS supplemented with 0.1% Triton X-100, Roche complete protease inhibitor cocktail (Roche), and CelLytic B (Sigma-Aldrich) at a ratio of 5 ml per gram of cell mass. Lysis proceeded on ice for 15 min, followed by centrifugation at 20,000 × *g* for 1 h to remove cell debris. Clarified lysates were then passed through a 0.22-μm filter and loaded onto a 1-ml Ni-NTA–agarose column that had been preequilibrated with 10 bed volumes of TBS. Loaded Ni-NTA–agarose was washed with TBS supplemented with 20 mM imidazole to reduce nonspecific protein binding. Finally, 6×His-NagK was eluted with 2 ml of TBS supplemented with 200 mM imidazole and dialyzed against 2 liters of TBS in 4 exchanges. The purity of 6×His-NagK was determined to be >95% based on gel staining with Coomassie brilliant blue R-250 (Acros).

Enzyme assays with 6×His-NagK were performed in kinase reaction buffer (40 mM Tris-HCl, 20 mM MgCl_2_, 0.1 mg/ml bovine serum albumin) using the ADP-Glo kinase assay (Promega) per the manufacturer's specifications. To each reaction, 20 μg of 6×His-NagK was added with various concentrations of ATP or substrate. Reaction progress was measured via luminescence using a BioTek Cytation 3 multimode plate reader in Perkin-Elmer 384-well AlphaPlates. Data analysis and plotting were performed using R and ggplot2 (62).

### Measurement of flux ratios.

Metabolic flux ratios were determined using the procedure described by Zamboni and coworkers with minor modifications (45). Glu^+^ strains of S. oneidensis were grown in 10 ml of minimal medium supplemented with either 100% [1-^13^C]glucose or a mix of 20% [U-^13^C]glucose/80% glucose (20 mM glucose total, Cambridge Isotope Laboratory) to mid-log phase (OD_600_, 0.3). Cells were harvested by centrifugation and were washed twice followed by resuspension in 500 μl of 6 M HCl. The cell resuspension was then heated to 100°C for 8 h followed by evaporation of the HCl under nitrogen flow for 4 h to yield dried cell hydrolysate. The dried hydrolysate was then gently resuspended in 100 μl of dimethylformamide. Subsequently, 40 μl of the crude hydrolysate was derivatized with an equal volume of *tert*-butyldimethylsilyl-*N*-methyltrifluoroacetamide with 1% *tert*-butyldimethylchlorosilane at 85°C for 1 h. The derivatized hydrolysate was injected into a Shimadzu QP2010 gas chromatograph/mass spectrometer (GC/MS) equipped with a 30-m Rxi-1ms column (Restek). The injection source was 230°C and the column was heated from 160°C to 310°C at 20°C min^−1^ during each experimental run. Metabolic flux ratios for key metabolites were determined by METAFoR analysis using the MATLAB FiatFlux analysis package (46). The data presented are the results from three biological replicates.

### Measurement of growth rates and yields.

Growth rates and yields were determined based on measurements of OD_600_ over time using an automated culturing system (63, 64). Cells were grown in minimal medium containing either lactate, glucose, or GlcNAc. Cultures were grown to saturation on these substrates followed by a 1:256 dilution into fresh medium, and 640 μl of each dilution was transferred to independent wells of a 48-well microtiter plate. Plates were incubated at 30°C and the OD_600_ was measured every 2 h for 48 h. OD data were corrected to media-only controls, and growth rates and yields were calculated using the CurveFitter software package. Each reported growth rate and yield value is the mean from 8 biological replicates.

### Accession number(s).

Sequencing data from this work have been deposited in the Sequence Read Archive under accession numbers SRR5359626, SRR5359627, and SRR5359628.

## Supplementary Material

Supplemental material
