# Supplementary material for: Growth Trade-Offs Accompany the Emergence of Glycolytic Metabolism in Shewanella oneidensis MR-1
Source: J Bacteriol. 2017 May 9;199(11):e00827-16. doi: 10.1128/JB.00827-16 (PMC5424254; doi:10.1128/JB.00827-16)
Supplement: Supplemental material [file supp_199_11_e00827-16__index.html]

Growth Trade-Offs Accompany the Emergence of Glycolytic Metabolism in Shewanella oneidensis MR-1 — Supplemental material 

# Growth Trade-Offs Accompany the Emergence of Glycolytic Metabolism in Shewanella oneidensis MR-1

## Supplemental material

- Supplemental file 1 -

  Fig. S1 (Purification of 6×His-NagK) and S2 (Growth and yield trade-offs of Glu+ and *nagR* mutants on lactate) and Tables S1 (Strains and plasmids) and S2 (DNA oligonucleotides)

  PDF, 318K
