# Supplementary material for: Growth Trade-Offs Accompany the Emergence of Glycolytic Metabolism in Shewanella oneidensis MR-1
Source: J Bacteriol. 2017 May 9;199(11):e00827-16. doi: 10.1128/JB.00827-16 (PMC5424254; doi:10.1128/JB.00827-16)
Supplement: Supplemental material [file JB.00827-16_zjb999094396s1.pdf]

Figure S1. Purification of 6xHis-NagK. Soluble protein from cell extract, column washes, and column elution displayed via 10% acrylamide Bis-Tris/MES-SDS gel electrophoresis. The gel was visualized by Coomassie Brilliant Blue R-250 staining.

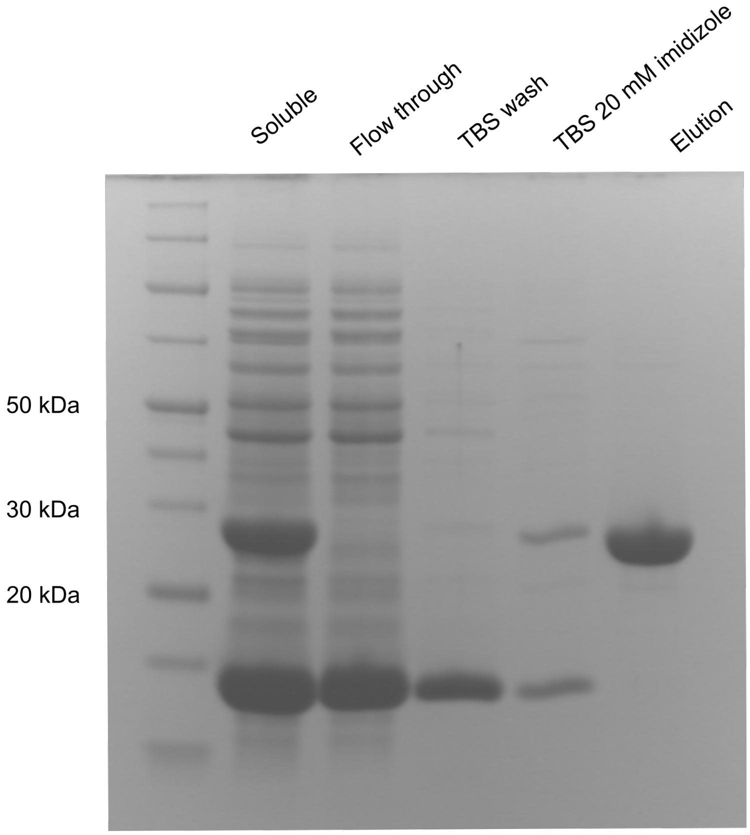

Figure S2. Growth and yield tradeoffs of Glu^{+} and *nagR* mutants on lactate. Wild-type and mutants were grown on 20 mM DL-lactate. Data are from at least 6 biological replicates. All mutants display a significant difference in growth rate and yield compared to wild-type (Wilcoxon rank sum test P<0.01).

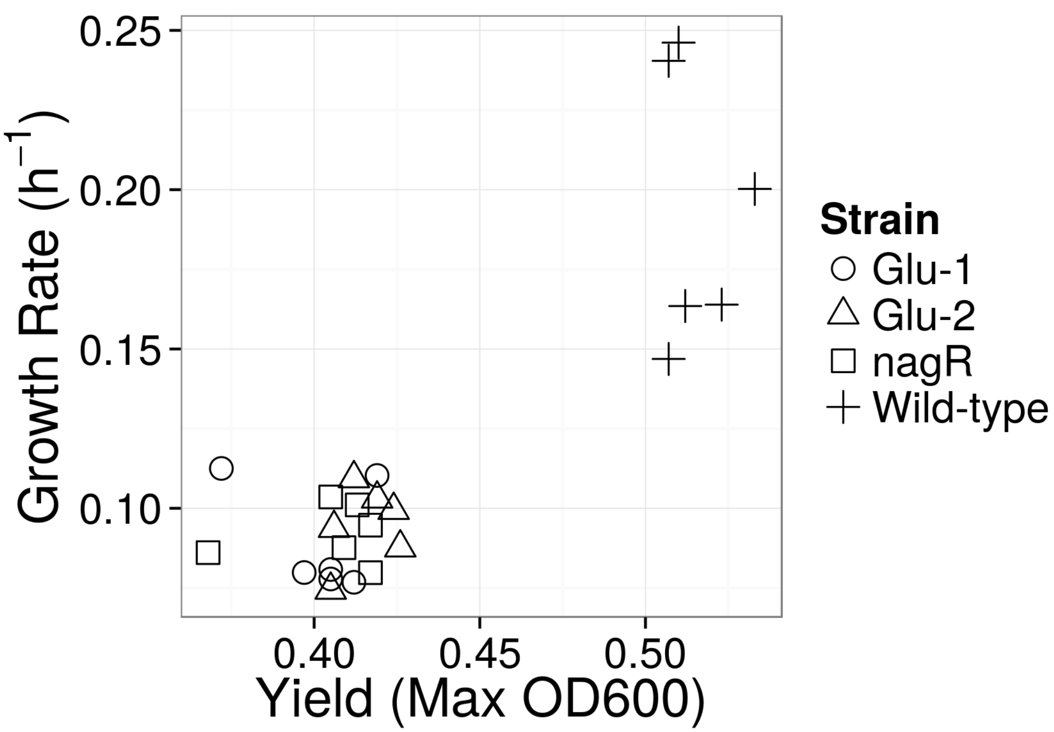

Table S1. Bacterial strains and plasmids used in this work.

Strain or plasmid	Relevant genotype	Source or reference
***Shewanella oneidensis* MR-1**		
S1419	ΔλSo ΔmuSoI ΔmuSoII	(1)
LC1650 (Glu-1)	S1419 Glu^+ Δ*hexB-pilY*	This work
LC1652 (Glu-2)	S1419 Glu^+ Δ*hexB-pilY*	This work
LC1765	S1419 Δ*nagR*	This work
LC1761	S1419 Δ*nagP*	This work
LC1762	LCH1650 Δ*nagP*	This work
LC1812	S1419 Δ*nagR ΔnagP*	This work
***Escherichia coli* K-12**		
NEB10β	*Δ(ara-leu)7697 araD139 fhuA ΔlacX74 galK16 galE15 e14- ϕ80*d*lacZ*ΔM15 *recA1 relA1 endA1 nupG rpsL rph spoT1 Δ(mrr-hsdRMS-mcrBC)*	New England Biolabs
BW25141	*ΔaraBAD567 ΔlacZ4787(::rrnB-3) Δ(phoB-phoR)580 galU95 ΔuidA3::pir recA1 endA9::FRT rph-1 ΔrhaBAD568 hsdR514*	(2)
WM3064	*thrB1004 pro thi rpsL hsdS lacZ*ΔM15 RP4-1360 *ΔaraBAD567 ΔdapA1341::[ermR pir]*	W. Metcalfe (University of Illinois)
ER2523	*fhuA2 [lon] ompT gal sulA11 R(mcr-73::miniTn10--TetS)2 [dcm] R(zgb-210::Tn10--TetS) endA1 Δ(mcrC-mrr)114::IS10*	New England Biolabs
Plasmids		

pBBR-MCS-2	*lacZα kan ori(pBBR) oriT*	(3)
pCM433	*cat sacB tetRA(RP4) bla ori(ColE1)*	(4)
pLC284a	*cat sacB ori(R6K) oriT*	This work
pQE80L	$P_{T5/lacO}$-6xHis *lacI*^{Q} *bla ori(ColE1)*	Qiagen
pPROBE-gfp	*rrnB(T1)x4 gfp kan ori(pBBR1) oriT*	(5)
pLC307	pPROBE-gfp::[*hexBp*]	This work
pLC311	pLC284a::[Δ*nagP*]	This work
pLC315	pLC284a::[Δ*nagR*]	This work
pLC318	pQE80L::*nagK*	This work
pLC319 (pNagR)	pBBR-MCS-2::*nagR*	This work
pLC321 (pNagP)	pBBR-MCS-2::*nagP*	This work

Table S2. DNA oligonucleotides used in this work.

Name	Sequence	Description
cat-sacB-F	ATA *ACG CGT* ATC TCC AGC AGC CGC AC	Amplification of *cat-sacB* from pCM433. Contains a *Mlu*I resistriction site.
cat-sacB-R	ATA *ACG CGT* GAT GGG TTA AAA AGG ATC G	Amplification of *cat-sacB* from pCM433. Contains a *Mlu*I resistriction site.
nagR-KO-1	GAC ATG GGA ATT CCC CTC CAC CGC GGT GGC CAC CAG GTT TAC CAA TAG ACT GAC	Amplification of the upstream region of *nagR*. Overhang homologous to pLC284a.
nagR-KO-2	TGA TCT AAA AAC TCT TTA TTC GAA TTT G	Amplification of the upstream region of *nagR*. Overhang homologous to downstream fragment.
nagR-KO-3	CAA ATT CGA ATA AAG AGT TTT TAG ATC AAG CGT GTT GAC GTT TTA AGA ATA TTT TTG	Amplification of the downstream region of *nagR*. Overhang homologous to upstream fragment.
nagR-KO-4	GCG TCC TCG GTA CCG GGC CCC CCC TCG AGG GAA GTT GCT GTG GGA GTG ATT GAT C	Amplification of the downstream region of *nagR*. Overhang homologous to pLC284a.
nagP-KO-1	GAC ATG GGA ATT CCC CTC CAC CGC GGT GGC GGC GTT TGT TTA GCT TTA GGT TGG	Amplification of the upstream region of *nagP*. Overhang homologous to pLC284a.
nagP-KO-2	TTC CAT AAA CCT GCT TCT TAT AGT AAT TAA	Amplification of the upstream region of *nagP*. Overhang homologous to downstream fragment.
nagP-KO-3	TTA ATT ACT ATA AGA AGC AGG TTT ATG GAA TAA AGC CAT AAA TGA CAC GAT CAC	Amplification of the downstream region of *nagP*. Overhang homologous to upstream fragment.
nagP-KO-4	GCG TCC TCG GTA CCG GGC CCC CCC TCG AGG GCA TGG GCG AGT AAT TTG GAC TA	Amplification of the downstream region of *nagP*. Overhang homologous to pLC284a.
nagR-pBBR-F	ATA *GAA TTC* CAA ATT CGA ATA AAG AGT TTT TAG ATC A	*nagR* amplification. Contains an *Eco*RI site.
nagR-pBBR-R	ATA *TCT AGA* CTA GTG CTT AGG TGA AGC CG	*nagR* amplification. Contains a *Xba*I site.
nagP-pBBR-	ATA *GAA TTC* TAC TAT AAG AAG	*nagP* amplification. Contains an *Eco*RI

F	CAG GTT TAT GGA A	site.
nagP-pBBR-R	ATA *TCT AGA* TTA TTT AGC AGA CCA ACT GCG	*nagP* amplification. Contains a *Xba*I site.
hexB-PROBE-F	ATA *GGT ACC* ATC TAA ACT AGA GAA CAC AGG GAT G	*hexB* promoter amplification. Contains a *Kpn*I site.
hexB-PROBE-R	ATA *GAA TTC* TAT ACT TTG CCA AAG CGG CG	*hexB* promoter amplification. Contains a *Eco*RI site.
nagK-QE80L-F	ATA GA*G GAT CC*G GAT TAG TCC AGA CAA ATG ATC	*nagK* amplification. Contains a *Bam*HI site.
nagK-QE80L-R	ATA *CTG CAG* TTA AAC TGT TGC TGA ATT AAA TTG C	*nagK* amplification. Contains a *Pst*I site.
pilY-F	GCG ATC CGT TGT GAC AAT G	*pilY* junction amplification.
pilY-R	TCC CGT TGC TTA TAC CGA AAC	*pilY* junction amplification.
3513-F	TGA TAA AAA TTA TGC TTA CCG TGA CG	SO_3513 junction amplification.
3513-R	AAA TTT GGC TCG CCA TTT GT	SO_3513 junction amplification.
RKO-1	CAG CGC TGC CGA GTA GAA TA	*nagR* deletion validation.
RKO-2	ACG ACA CCT CTC GGA CTT CT	*nagR* deletion validation.
PKO-1	ACG AAT GCC TGA TCC TGA AG	*nagP* deletion validation.
PKO-2	GCA AAT GGG AGA AAT ACC GTG	*nagP* deletion validation.

References

1. **Gödeke J**, **Paul K**, **Lassak J**, **Thormann KM**. 2011. Phage-induced lysis enhances biofilm formation in *Shewanella oneidensis* MR-1. ISME J **5**:613–626.

2. **Haldimann A**, **Wanner BL**. 2001. Conditional-replication, integration, excision, and retrieval plasmid-host systems for gene structure-function studies of bacteria. J Bacteriol **183**:6384–6393.

3. **Obranić S**, **Babić F**, **Maravić-Vlahoviček G**. 2013. Improvement of pBBR1MCS plasmids, a very useful series of broad-host-range cloning vectors. Plasmid **70**:263–267.

4. **Marx CJ**. 2008. Development of a broad-host-range *sacB*-based vector for unmarked allelic exchange. BMC Res Notes **1**:1.

5. **Miller WG**, **Leveau JHJ**, **Lindow SE**. 2000. Improved *gfp* and *inaZ* Broad-Host-Range Promoter-Probe Vectors. Mol Plant Microbe Interact **13**:1243–1250.
